# Comparative analysis of energy transfer mechanisms for neural implants

**DOI:** 10.3389/fnins.2023.1320441

**Published:** 2024-01-16

**Authors:** Sols Miziev, Wiktoria Agata Pawlak, Newton Howard

**Affiliations:** ni2o Inc., Washington, DC, United States

**Keywords:** brain implants, energy transfer, wireless power transmission, brain computer interfaces, inductive coupling, capacitive coupling, ultrasound power transmission, optical energy transfer

## Abstract

As neural implant technologies advance rapidly, a nuanced understanding of their powering mechanisms becomes indispensable, especially given the long-term biocompatibility risks like oxidative stress and inflammation, which can be aggravated by recurrent surgeries, including battery replacements. This review delves into a comprehensive analysis, starting with biocompatibility considerations for both energy storage units and transfer methods. The review focuses on four main mechanisms for powering neural implants: Electromagnetic, Acoustic, Optical, and Direct Connection to the Body. Among these, Electromagnetic Methods include techniques such as Near-Field Communication (RF). Acoustic methods using high-frequency ultrasound offer advantages in power transmission efficiency and multi-node interrogation capabilities. Optical methods, although still in early development, show promising energy transmission efficiencies using Near-Infrared (NIR) light while avoiding electromagnetic interference. Direct connections, while efficient, pose substantial safety risks, including infection and micromotion disturbances within neural tissue. The review employs key metrics such as specific absorption rate (SAR) and energy transfer efficiency for a nuanced evaluation of these methods. It also discusses recent innovations like the Sectored-Multi Ring Ultrasonic Transducer (S-MRUT), Stentrode, and Neural Dust. Ultimately, this review aims to help researchers, clinicians, and engineers better understand the challenges of and potentially create new solutions for powering neural implants.

## 1 Introduction

The escalating incidence of neurological conditions, which have become the top contributor to global disability and the second most common reason for mortality, is fueling significant expansion in the market for brain implants (Dumurgier and Tzourio, 2020; Feigin et al., 2020). This trend is further amplified by an increasingly aging global demographic (Burden of neurological conditions, n.d.). As of 2023, the global valuation for the brain implant market stood at approximately USD 6.4 billion (Brain Implants Market Insights, n.d.; Brain Implants Market Size, n.d.), with projections estimating a rise to USD 11.02 billion by 2028 (Brain Implants Market Insights, n.d.). In this context, “brain implants” are defined as any technological devices that establish a direct interface with the biological brain of a subject.

In the face of this surge in neurological disorders, brain implants have emerged as a crucial therapeutic avenue. They have shown significant efficacy in improving the quality of life for those suffering from a range of conditions, including but not limited to Parkinson’s disease, ALS (Willett et al., 2023), movement disorders, epilepsy, and mental health issues (Fisher and Velasco, 2014; Bergfeld et al., 2016; França et al., 2022). These devices afford more precision in modulating neural circuits than non-invasive techniques, enabling targeted treatments and interventions.

Yet, the transformative potential of brain implants is hindered by significant technical challenges, most notably in power sourcing and power transfer. The lifespan of implants can vary widely following surgical implantation, largely due to differences in power sources and delivery mechanisms. Non-rechargeable fully implanted devices, like some pacemakers, are powered by built-in batteries and do not require external charging. These devices have a limited lifespan as they need surgical replacement once batteries deplete (Nelson et al., 2020). However, our paper does not cover non-rechargeable fully implanted devices, focusing on energy transfer mechanisms for neural implants not energy storage. Another innovative area of interest is the development of battery-less, self-harvesting implants. These devices use energy from the body’s biological processes or external sources, such as kinetic, thermal energy, or utilizing glucose in biofuel cells, to function without the need for an internal or external battery (Bullen et al., 2006; Rapoport et al., 2012; Amar et al., 2015; Owida et al., 2021; Shuvo et al., 2022). This approach represents a significant advancement in minimizing surgical interventions for battery replacements, thus reducing the biological impact on tissues. Despite the innovative nature of these self-powered devices, this paper does not review their mechanisms and applications. Our focus remains primarily on the challenges and advancements in currently utilized energy transfer methods for brain implants that rely on traditional power sources and not self-powering devices. The majority of current implants predominantly rely on wireless-inductively rechargeable batteries for power. Battery longevity also varies extensively, affected by patient diagnosis, implant parameter settings—such as the duration and frequency of stimulation and simultaneous recording capabilities—as well as individual therapeutic needs. For instance, fixed-life batteries in Deep Brain Stimulation (DBS) generally last between 3 to 5 years (Qiu et al., 2021; Wang et al., 2021). Some non-rechargeable batteries can extend this to 6–7 years or beyond (Hariz, 2019) while rechargeable neurostimulators have been reported to last up to 15 years (Medtronic, n.d.-a; Vercise DBS Product Details | Boston Scientific, n.d.).

In contrast, battery-free implants like ni2o’s KIWI use wireless power transfer and are smaller, enabling placement in new locations (Howard, 2017). KIWI utilizes wireless connectivity and inductive charging, aligning with previously discussed electromagnetic methods. The implant features architecturally complex carbon nanotube (CNT) electrodes which enable high spatial resolution for targeted neural stimulation, allowing the implant to focus energy solely on malfunctioning neurons. This localized approach significantly reduces overall energy consumption while circumventing the need for external batteries by incorporating a supercapacitor approach. Another example of a battery-free implant is the Stentrode, designed for a 10-year lifespan, avoids invasive battery replacements through wireless power using near-field RF, with a transmission depth of ≈30 mm and 2% efficiency, connected to an Inductively Powered Internal Telemetry Unit (ITU) for power and Bluetooth signal transmission (Aldaoud et al., 2018; Oxley et al., 2020; Raza et al., 2020). Similarly, the Utah Microelectrode Array (UEA) uses an external power mechanism, offering longevity with some lasting over 2.7 years, and even one case up to 9 years (Sponheim et al., 2021). These examples illustrate the trade-offs in brain implant technologies between internal and external power sources, balancing longevity, invasiveness, and external hardware practicality, each impacting patient care and life quality (Hochberg et al., 2006; Kim et al., 2008, 2011).

## 2 Complexities and challenges in powering brain implants

Energy constraints are a significant hurdle, especially in fully-implantable systems where the energy source must be compact yet capable of sustaining the implant’s functions over extended periods (Rosidi et al., 2011). Biocompatibility requires the materials and techniques to be safe for long-term interaction with biological tissues. Furthermore, the inter-relationship between power transfer and data communication in these implants introduces additional layers of difficulty. This relationship often leads to developed techniques and strategies that aim to balance efficient power delivery with effective data transmission. However, these solutions can sometimes result in compromises, such as trade-offs between the size of the implant and its power capacity or between the energy efficiency and the data rate (Santiago and Westerink, 1990; Deboer and Abercrombie, 1996; Jaquins-Gerstl and Michael, 2009; Kozai et al., 2012).

### 2.1 Energy constraints

#### 2.1.1 Limited options for power supply

Current technologies fall short of offering a perfect alignment between the brain’s biology and the biocompatibility of implantable devices. Each procedure inherently carries its own set of risks; scientists merely aim to minimize these hazards by carefully selecting power sources and technologies that pose the least long-term risk, all while holding the belief that the therapeutic advantages outweigh the downsides.

The limitation in available power supply options poses a significant challenge in powering brain implants, often restricting the device’s key features such as power density, including its diagnostic and therapeutic capabilities, operational lifespan, and duty cycle (Yang et al., 2021). This constraint emanates from a complex interplay of factors such as durability, energy requirements, size constraints, biocompatibility considerations, and the tradeoff between requisite power supply and feasible energy delivery mechanisms to minimize surgical interventions.

Moreover, the relationship between power source types and an implant capability is not linear, yet there are visible patterns: implants with a greater number of channels and more complex functionalities (e.g., simultaneous recording and stimulation) exhibit a propensity for higher energy demands. Consequently, these tend to rely on battery-based energy sources as opposed to supercapacitors or wireless energy transfer methods (Table 1).

**Table 1:** Overview of leading brain implant technologies.

Company/Research group	Implant name	Energy source	Number of channels	Functionalities	References
Neuralink	N1	Inductively rechargeable battery	3,072	Electrical recording, electrical stimulation	Musk and Neuralink (2019)
Medtronic	Activa SC	Non-rechargeable battery	1	Electrical stimulation	Medtronic (n.d.-b)
Medtronic	Activa RC	Inductively rechargeable battery	2	Electrical recording, electrical stimulation	Medtronic (n. d.-c)
Boston scientific	Vercise Genus P16	Inductively rechargeable battery	2	Electrical stimulation	DBS Product Details-Vercise Genus DBS System (n.d.)
Abbott (St. Jude)	Infinity DBS	Non-rechargeable Battery	16	Electrical stimulation	InfinityTM system for deep brain stimulation (n.d.)
Blackrock microsystems	Utah array	Hardwired	1,024	Electrical recording, electrical stimulation	Blackrock Neurotech (2023)
Synchron	Stentrode	Inductive powering	16	Electrical recording, electrical stimulation	Mitchell et al. (2023)
Brains in Silicon group Stanford	NeuroGrid array	Hardwired	120–256	Electrical recording	Khodagholy et al. (2017), Kashkoush et al. (2019)
UC Berkeley	Ultrasonic neural dust mote	Ultrasonic powering	1	Electrical recording	Seo et al. (2015)
ni2o inc.	KIWI	Inductive powering	10,000−100,000	Electrical and optical recording, electrical, and optical stimulation	Howard (2017)

#### 2.1.2 Energy requirements

Brain implants present a unique set of challenges when it comes to energy needs, requiring a balance between functionality and safety. These devices perform a wide range of operations—data acquisition, processing, stimulation, and telemetry to external devices—all of which draw on the implant’s power resources. The power needs vary depending on the specific application, design, and system architecture of the implant. Of particular note is the energy-intensive nature of the processing and recording circuitry, attributed to the high transistor count necessary for tasks like data processing and filtering. However, these demanding energy requirements are tempered by regulatory guidelines. Agencies such as the FDA as well as their European counterparts, impose stringent limitations on energy consumption to ensure patient safety. For example, brain-computer interfaces (BCIs) are subject to federal and international guidelines that cap their power usage at 15–40 mW, with the exact limit varying based on the implant’s depth within the brain tissue (Rapeaux and Constandinou, 2021; Yale Engineering Magazine, 2021).

The temperature difference between an implant and surrounding tissue is a critical safety metric, since power usage in the implant converts into heat, risking overheating and potential brain tissue damage (Wolf, 2008). Consequently, implants must adhere to FDA and IEEE standards for electromagnetic disturbances (Center for Devices and Radiological Health, 2021). To ensure safety, these implants often utilize low-power circuitry, which, while limiting computational capabilities and data throughput, is essential. To compensate, computational tasks are typically offloaded to an external computer, mitigating the implant’s power consumption (Darwish and Hassanien, 2011).

#### 2.1.3 Trade-offs in data rate and power consumption

Designing brain implants involves trade-offs between data rate and power consumption. Research has highlighted the importance of considering the neural interface architecture and the trade-offs between power consumption and complexity in the design of wireless intracortical brain computer interfaces (iBCIs) (Even-Chen et al., 2020). For example, the number of recording channels in iBCIs is limited by the power budget of the implantable system, and designing for lower bit error rate (BER) can improve power consumption (Even-Chen et al., 2020). Additionally, there is a fundamental trade-off between power transfer efficiency and spectral efficiency in inductive links for biomedical implants (Dehghanzadeh et al., 2021). Power consumption is a significant concern in the design of neural interfaces, especially for battery-powered implantable applications, as it can impact the lifetime of the implants and the resources on-chip (Zhang et al., 2022). Therefore, when designing brain implants, careful consideration of the trade-offs between data rate and power consumption is essential to ensure optimal performance and longevity of the implantable systems.

### 2.2 Physical constraints in power supply components

The physical constraints related to the power supply within brain implants are further influenced by the component’s shape, size, and thermal properties (Wolf, 2008). These constraints can significantly affect the quality of signals from sensors implanted in the central nervous system, both in the short term (seconds to minutes) and over extended periods (weeks to months).

#### 2.2.1 Geometry and heat dissipation

The geometric configuration of the energy storage component—be it a battery, capacitor, or other form—might play a critical role in both anatomical compatibility and thermal management. Limited research exists on the thermal properties of batteries in neural implants, but studies on lithium-ion batteries in automotive applications offer valuable insights. For instance, research on much larger electric vehicle batteries has demonstrated that different geometries significantly influence heat dissipation and internal temperature variations (Tendera et al., 2022; Dubey et al., 2023). Such findings suggest that the geometry of the battery could be an important factor in managing thermal risks in neural implants considering the micro scale of the neural environment. To mitigate the risk of thermal damage, these devices must strictly limit temperature increases in surrounding tissues to less than 0.5°C, all while maintaining sufficient power output for device functionality (Wolf, 2008; Nurmikko, 2020; Rapeaux and Constandinou, 2021).

#### 2.2.2 Power consumption and its thermal implications

While the discussion so far has largely focused on geometry’s role in thermal management, it is imperative to explore how specific operational demands can exacerbate thermal constraints together with geometry of the power receiving unit. The number of recording sites, the sophistication of the signal processing chain, and the need for telemetry and transcutaneous energy delivery all push the power consumption of an implanted device toward a point where thermal burden becomes a limiting factor. Existing research and guidelines shed light on this (Wolf, 2008).

The heat from the implanted system must be adequately dissipated to prevent an adverse tissue response. Industry guidelines suggest that a 2°C temperature increase, a 40 heat flux, and a 1.6 mW/g power dissipation are reasonable limits for implanted devices (Wolf, 2008). By focusing on a subset of brain waves, researchers have dramatically reduced the power requirements of neural interfaces while improving accuracy. Nason et al. (2020). Simulations from another study indicated that the skull unit (SU) implant could operate at a maximum power of 75 mW without causing the temperature of the adjacent tissues to exceed the established safety threshold (Serrano-Amenos et al., 2020).

#### 2.2.3 Energy density and storage modalities

The advancements in battery technology are not merely about making technology smaller or more flexible but also about enhancing their ability to store more energy in a given volume over a longer period of time, which is the essence of energy density. Yet, this potential is often untapped as traditional rigid batteries continue to dominate implant designs. These conventional batteries, although energy dense, consume over half of the implant’s total volume and have limited duration of power supply (Yang et al., 2021). This volume constraint intensifies the need for more energy-dense, yet compact, alternatives.

Conventional batteries, often bulky and rigid, are difficult to reshape due to their composite electrodes and liquid electrolytes. Recent research has been focused on developing new electrode and electrolyte materials to facilitate the creation of flexible, low-profile, or micro-sized batteries without compromising energy density. A significant advancement in this area has been the development of solid-state electrolytes, allowing the thickness of these microbatteries to be reduced to mere micrometers. These are typically composed of thin-film solid-state materials like polymers, silicon, or carbon pillars, and can be fabricated using thick-film technology or vapor deposition (Nie et al., 2018; Yang et al., 2021; Salado and Lizundia, 2022).

While energy density primarily refers to the amount of energy that can be stored per unit volume, flexibility can allow for more efficient use of available space within the constrained environment of a medical implant. Flexible batteries have gained traction in diverse applications, from smartphones to wearable healthcare devices (Yang et al., 2021). Notably, the J. Flex battery by Jenax is a lithium-ion polymer battery that can be twisted, bent, and folded, making it highly suitable for medical devices. With a market size of $98 million in 2020 and expected to grow to $220 million by 2025, flexible batteries are becoming increasingly important (Kong et al., 2020).

#### 2.2.4 Miniaturization

Power density is crucial in brain implant design, linking performance to the implant’s size, which must be minimized to reduce biomechanical stress on surrounding neural tissue. High power density, constrained by size, is essential to avoid tissue damage and foreign body responses, which can further affect the signal quality across all central nervous system sensors over both acute and chronic time frames (Bazaka and Jacob, 2012; Kozai et al., 2015). The implant’s size and volume directly correlate with the risk of intracerebral hemorrhage and ischemic injuries (Rosidi et al., 2011), with larger implants exerting more pressure, potentially leading to vascular compression and secondary injuries (Jaquins-Gerstl and Michael, 2009; Kozai et al., 2012). Conversely, smaller devices, like carbon fiber electrodes, show reduced tissue damage and inflammatory responses while maintaining better signal quality (Santiago and Westerink, 1990; Deboer and Abercrombie, 1996).

The power unit’s size is a balancing act between being small enough for comfortable implantation and providing sufficient energy for the implant’s lifespan. Batteries, for instance, can occupy up to 90% of an implant’s volume and 60% of its weight (Won et al., 2021), with space and thermal output being major constraints (Rapeaux and Constandinou, 2021). Wireless power transmission advancements could reduce the need for bulky batteries (McGlynn et al., 2021).

Modern implants are miniaturized for biocompatibility, with examples like Neural Dust and borosilicate glass-encapsulated implants demonstrating tiny dimensions (Patch, 2021). Designing energy sources for such small scales without sacrificing functionality is challenging. Implants must be hermetically sealed and have a regulated density; for instance, one study achieved a density about twice that of brain tissue without adverse tissue reactions or migration (Dabbour et al., 2021). No adverse tissue reactions or migration tracks were observed in the study, suggesting effective biocompatibility. The ideal density for an implant is close to 1 g/cm^3^ to minimize glial scarring and optimize functionality (Lind et al., 2013), though density requirements can vary based on brain region, patient, and implant material.

#### 2.2.5 Durability

The power source must provide a stable energy output throughout its lifespan to ensure consistent performance. Both the device and the power source must be durable enough to last for several years without requiring frequent replacements. These energy demands must be consistently met without failure or significant fluctuation in order to ensure continuous monitoring and stimulation in a timely manner depending on the purpose of the implant. For instance, implants that restore vision through wireless charging, such as the Intracortical Visual Prosthesis (ICVP) which circumvents the retina and optic nerves to directly interface with the brain’s visual cortex, require uninterrupted energy supply to sustain the patient’s sight (A Phase, 2020). Another study focusing on battery drain in Deep Brain Stimulation (DBS) found that charge density and total power were significantly related to power source life (Fakhar et al., 2013). The study observed clinical worsening in 38 cases, which improved following battery replacement. This suggests that power delivery either by battery or other wireless method not requiring battery can significantly impact symptom severity and should be closely monitored. The study used both the University of Florida (UF) estimator and the Medtronic helpline to estimate battery life, both of which were significantly correlated with actual battery life (Fakhar et al., 2013).

### 2.3 Biocompatibility

#### 2.3.1 Tissue reaction to material density

The density of an implant is a multifaceted parameter, influenced not only by its geometrical design but also by the choice of materials for both the structural components and the power source (Saini, 2015). For instance, lithium-ion batteries, commonly used in implants, have a material density ranging from 0.534 to 3.5 g/cm^3^, depending on the specific composition of the cathode and anode materials (Lin et al., 2017; Li, 2019; Karabelli and Birke, 2021) contributing significantly to the implant’s overall density affecting its biocompatibility and operational efficiency within the neural tissue.

On the other hand near field radio-frequency energy harvesting system commonly constructed from materials like polydimethylsiloxane (PDMS) with a density of approximately 0.97 g/cm^3^ (PDMS, n.d.; Samiee et al., 2019) or supercapacitors, often fabricated from materials such as carbon aerogels with densities ranging from 0.182 to 0.052 g/cm^3^ (Feng et al., 2011) or according to new studies even as low as 0.16 g/cm^3^ (Yanagi et al., 2021) allowing for much lighter construction of the entire brain implant thereby potentially reducing biomechanical stress and the subsequent inflammatory reaction within the brain tissue both in long and short term.

#### 2.3.2 Physiological safety

The safety implications can be broadly categorized into two types: thermal and non-thermal effects. Thermal effect will be further discussed in later sections. Biocompatibility is a cardinal requirement for the power sources used in neural implants, necessitating rigorous standards and guidelines to ensure physiological safety. Regulatory bodies such as FDA, as well as European Agencies, provide comprehensive frameworks for such assessments.

FCC classifies electromagnetic emissions into two categories: ionizing and non-ionizing. Implantable medical technologies usually operate in the non-ionizing category to reduce the likelihood of causing molecular alterations in biological tissues In the U.S., the Federal Communications Commission (FCC) allocates specific frequency spectrums for medical applications. For example, a 24 MHz spectrum in the 413–457 MHz range is designated for Medical Micropower Networks (MMNs), benefiting brain implant technologies (Mahn, 2013). Furthermore, the FCC has earmarked the 401–406 MHz bands for Medical Device Radio Communication Service (MDRC), with varying channel bandwidths between 100 and 300 kHz (Hardell and Sage, 2008; Stam, 2018; Soliman et al., 2021). These allocations are particularly suited for wearable and implantable medical devices that require moderate data transfer rates (Nelson et al., 2020).

Safety standards are also set by organizations like the International Commission on Non-Ionizing Radiation Protection (ICNIRP) and the Institute of Electrical and Electronics Engineers (IEEE) (SCENIHR, 2008) These entities aim to minimize bio-effects such as tissue heating by enforcing Specific Absorption Rate (SAR) limits which accounts for tissue electrical conductivity, electric field intensity, and tissue mass (Chiao et al., 2023). The IEEE’s C95.1 Standard, for instance, limits the 10-g averaged SAR to 1.6 W/kg over a 6-min period. However, some studies (Bocan et al., 2017) suggest the need for more stringent SAR guidelines for implantable devices as conventional methods for estimating SAR might underestimate the actual absorption levels due to factors like the body’s heat dissipation mechanisms, variations in tissue conductivity and density. Studies also mention that temperature changes are influenced not just by SAR but also by factors like heat conduction, blood perfusion, and metabolic heat generation rates which might be hazardous in sensitive neural tissue.

Moreover, the FDA requires a thorough evaluation of the medical device in its final, sterilized form. Their biocompatibility assessment includes a categorization based on the implant’s contact surface—be it neural tissue, bone, cerebrospinal fluid (CSF), or blood. For instance, intracortical electrodes, which are implanted in the brain’s cortex, must meet specific biocompatibility endpoints. These include but are not limited to cytotoxicity, sensitization, irritation or intracutaneous reactivity, acute systemic toxicity, material-mediated pyrogenicity, and neurotoxicity. While these standards are primarily aimed at the overall brain implant, it is imperative that both the power source and the power delivery methods adhere to these biocompatibility criteria (Implanted brain-computer interface (BCI) devices for patients with paralysis or amputation - non-clinical testing and clinical considerations, 2021; Center for Devices and Radiological Health, 2023).

#### 2.3.3 Biochemical interaction

Despite the prevalent use of biocompatible encapsulating materials such as polyimide or parylene for brain implants, the risk of battery leakage remains a critical concern. Such leakage could potentially release substances like lithium salts or other electrolytes into the neural environment, emphasizing the necessity for fault-tolerant designs and monitoring (Lu et al., 2020; Chen et al., 2021).

For instance, cytotoxic effect may result from the cellular uptake of leaked substances, leading to necrosis or apoptosis. Moreover, these chemicals can elevate reactive oxygen species levels, inducing oxidative stress by increasing the concentration of reactive oxygen species (ROS) that may damage cellular DNA, proteins, and lipids (Zhang, 2018). Additionally, an acute inflammatory response might be triggered, activating microglia, the resident immune cells in the brain, to produce cytokines and free radicals that could further harm neural cells (Pei et al., 2016). These leaked substances can also disrupt the ionic balance of essential elements like sodium, potassium, and calcium, leading to excitotoxicity—a pathological process causing neuron damage due to excessive neurotransmitter stimulation. Further complications can arise from altered cellular metabolism, affecting energy balance within neurons and potentially causing cell death. Lastly, in response to chemical exposure, neural tissue might undergo fibrosis, a defensive yet functionally compromising mechanism (Grisold and Carozzi, 2021; Schander et al., 2021; Yin et al., 2021; World Health Organization: WHO, 2023).

#### 2.3.4 Heat impact on tissues

Effective thermal management is crucial for neural implants, given the brain’s unique sensitivities to factors like temperature, tissue interactions, and biocompatibility. Although Li/I2 batteries have been successfully used in cardiac pacemakers due to their high discharge voltages reaching up to 3.6 V and impressive energy densities of 210 and 810 W h/L, they are not directly applicable to brain implants (Zebda et al., 2018).

Studies on rat models reveal that brain temperature is inherently unstable, fluctuating between 2 and 4°C under normal physiological and behavioral conditions. Furthermore, physiological hyperthermia in the rat brain appears to be adaptive under normal conditions, enhancing neural functions (Kiyatkin, 2019). Such thermal variations could adversely affect neural activity, homeostatic parameters, and cellular integrity if augmented by additional heat. Importantly, these findings are based on animal studies, and extrapolation to the human brain requires caution. The rat model does not directly discuss the impact of brain implant on additional heat generation; however, they further discuss it in the light of chemically induced temperature changes such as those caused by drugs which can exceed beyond its upper physiological limit disrupting thermal dynamics leading to maladaptive neural activity and life-threatening complications (Kiyatkin, 2019).

In humans, brain temperature regulation adds layers of complexity to the thermal management of neural implants. The brain’s core temperature can be up to 2°C higher than body temperature, and variances of 0.5–1°C exist between the brain’s center and surface (Wang et al., 2014; Gowda et al., 2018). As a review by Wang et al. (2014) summarizes, most of the processes within the brain show sensitivity to temperature changes. These temperature sensitivities necessitate stringent thermal controls to avoid adverse impacts on neuronal metabolism and functionality (Gowda et al., 2018).

As previously discussed in the safety section, conventional methods for estimating Specific Absorption Rate (SAR) may underestimate the actual heat absorption levels according to some studies (Bocan et al., 2017). This is especially crucial given the brain’s intrinsic thermoregulatory mechanisms, including the cerebrospinal fluid, intracranial blood flow, and capillary networks that act as thermal buffers (Wang et al., 2014). One of the mentioned factors, heat dissipation might lead to underestimate the actual absorption levels possessing additional risk for the neural environment. This is particularly relevant in neural tissues where lower blood flow can lead to greater temperature fluctuations, highlighting the limitations of SAR as a direct predictor of tissue temperature. The bioheat equation mentioned in the study further elucidates the intricate balance between heat accumulation and dissipation in tissues, incorporating variables such as heat conduction, blood perfusion, and microwave heating.

Adding another layer of complexity are specialized neurons in the preoptic anterior hypothalamus (POA), which are central to the brain’s thermal sensitivity. These neurons play a key role in physiological responses to temperature changes as most of these neurons are sensitive to warmth, increasing their activity as temperature rises triggering further regulations (Romanovsky, 2018). Moreover, some molecules, such as transient receptor potential (TRP) channels like TRPM2, are known to act as heat sensors in hypothalamic neurons (Romanovsky, 2018). While neurons responsive to cold temperatures also exist, they are less common and generally activated indirectly by warmth-sensitive neurons, emphasizing the greater risks associated with overheating as opposed to overcooling (Romanovsky, 2018).

The peripheral sensory neurons, mostly sensitive to cold, relay deep body temperature information to the brain. This is particularly crucial given that the majority of central thermoreceptors are warmth-sensitive. For an in-depth discussion of the thermal effects on the brain, consult the referenced article which covers topics ranging from dopamine regulation to tissue-level sequelae like parenchymal edema and damage to the blood–brain barrier (Wang et al., 2014).

Recharging is another potential issue to consider. Recharging neural implants via an external skin-mounted antenna can increase temperature, by creating eddy currents and Ohmic heat within the implant. This process, observed in systems like Medtronic Restore and ANS Eon, can significantly raise localized tissue temperature (Lovik et al., 2009). Prolonged recharging may even lead to tissue necrosis, especially if heat dissipation at the skin surface is hindered.

### 2.4 Interrelationship between power transfer and data transfer

#### 2.4.1 Interference between power and data signals

Interference between power and data signals presents a significant challenge in neural implant design, especially in fully-implantable devices with rechargeable batteries. Wireless charging methods like inductive coupling, while efficient for power delivery, can cause electromagnetic interference (EMI) with RF-based data transmission, disrupting communication integrity (Seckler et al., 2015; Driessen et al., 2019; Nadeem et al., 2021).

Battery-less implants, such as those using supercapacitors, depend on frequent and efficient power transfer like near-field communication (NFC) or RF. These methods, while effective for power delivery, can conflict with the frequencies used for data transfer resulting in signal corruption, which can affect the diagnosis and monitoring of neurological conditions (Dobkin, 2012; Mattei et al., 2016).

The main challenge is managing efficient power transfer alongside reliable data communication within limited physical and electromagnetic spaces. Proximity in circuits or spectrum can lead to signal interference. For example, RF systems for charging may induce voltages in nearby data circuits, causing data loss or errors, while power harvesting in battery-less systems might disrupt high-frequency data signals (Bazaka and Jacob, 2012; Rahimpour et al., 2021; Won et al., 2021).

To mitigate interference, one strategy is using separate frequency bands for power and data. Lower frequencies are reserved for inductive charging and higher ones for RF communication, minimizing interference risks (Kainz et al., 2003; Dionigi and Mongiardo, 2012; Degen, 2021). Time-division multiplexing is another approach, alternating between power and data transmission to avoid simultaneous interference, though it adds complexity and may reduce efficiency (Åkesson et al., 2015; Jiang et al., 2017; Holzapfel and Giagka, 2022). Additionally, novel shielding techniques and electronic filters can isolate power circuits from data circuits, reducing cross-interference. However, this solution increases design complexity and could impact the implant’s size, a critical factor in miniaturization (Amali et al., 2021; Wang Z. et al., 2022).

#### 2.4.2 Cross-talk and signal integrity

Cross-talk and signal integrity in neural implants pose distinct challenges compared to the interference issues discussed earlier. While interference predominantly deals with external sources affecting the implant’s operation, cross-talk is an internal challenge of unwanted coupling of signals between different circuits or channels within the implant itself. This internal interference can be particularly problematic in the compact environments of neural implants potentially distorting the signal and leading to data loss or errors (McNamara et al., 2021).

For instance in fully-implantable devices with rechargeable batteries, the integration of power charging circuits with data transmission circuits in a limited space can lead to cross-talk. The switching regulators used for power management might induce noise in adjacent data communication lines. Battery-less implants, such as supercapacitor type implants, on the other hand, often rely on circuit designs to manage frequent power harvesting and data transmission (Gall et al., 2018). The close proximity of these circuits increases the risk of cross-talk, where the power harvesting circuit might interfere with data signal integrity. Two-part systems with external connections often combine wired power supply with wireless data communication.

One effective way to reduce cross-talk is by physically separating power and data lines within the implant and using shielding techniques (Köse et al., 2011). This approach, however, can increase the size of the implant and may pose challenges for miniaturization efforts.

## 3 Existing methods of delivering power to implants

**Figure 1:**
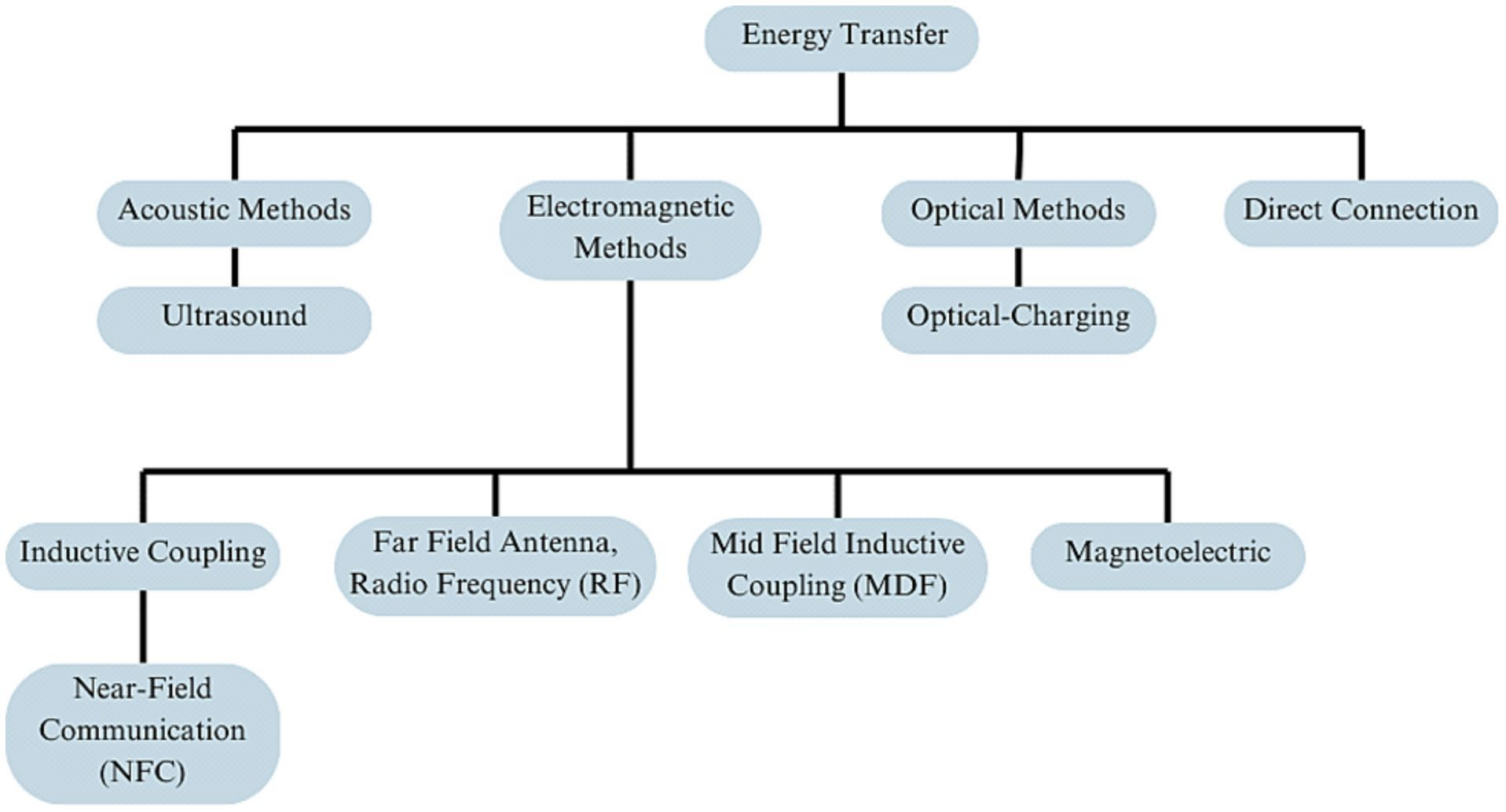
Overview of energy transfer methods in neural implant technology, organized into four primary branches: acoustic methods, electromagnetic methods, optical methods, and direct connection.

Biomedical implantable devices (BIDs) increasingly utilize various methods for energy transmission to overcome the need for frequent battery replacement. These include wireless options like Inductive Coupling, Ultrasound, NFC, and Optical methods, as well as wired solutions like Hardwired Direct External Connections (Figure 1). The transmitted energy is typically stored in internal rechargeable batteries or capacitors (Mendoza-Ponce et al., 2018), even soft ones (Li et al., 2019; Sheng et al., 2021) for sustained use, although some systems may offer continuous power supply directly to capacitors. Wireless options offer enhanced patient mobility but face challenges like low energy absorption and limited transmission range (Table 2). In contrast, wired methods like Direct External Connections do not face these challenges yet require implants outside of the skull. As of 2020, approximately 10% of medical implants employed some form of wireless power, with Inductive Coupling being the most commonly used.

**Table 2:** Comparison of power transferring methods for brain implant technologies.

Power transfer method	Efficiency	Transmission distance	Operating frequency (MHz)	Safety concerns	Challenges	Optimization techniques	Citation
Inductive coupling	High	mm − cm	MHz − GHz	Device overheating, tissue damage	Coil misalignment	Coupling coefficient, machine learning	Al-Kalbani et al. (2012), Freeman and Byrnes (2019)
Capacitive coupling	Medium	mm − cm	kHz − MHz	Electrical interference	Energy storage	Coupling coefficient, coupling capacitance	Sodagar and Amiri (2009), Jegadeesan et al. (2017), Narayanamoorthi (2019)
Radio frequency (RF)	Medium	cm − m	MHz − GHz	Depending on the range−cancer risks	Antenna size, tissue properties	*In-vivo* networking, beamforming algorithms	Ahn and Hong (2014), Fan et al. (2019), Narayanamoorthi (2019), Iqbal et al. (2022), Radiofrequency (RF) (n.d.)
Near-Field communication (NFC)	Low	cm	13.56	Data corruption or modification, tissue absorption	Antenna size, Return loss	Direct antenna modulation (DAM), phase shift keying (PSK)	Shin et al. (2017), Biswas et al. (2018), He et al. (2018)
Ultrasonic	Low	cm	kHz − MHz	Tissue absorption	Receiver misalignments	Beamforming, S-MRUT	Seo (2013), Santagati et al. (2020)
Optical charging	Very low	−	THz	Tissue absorption, Thermal effects	Tissue opacity, scattering	Reflector, photodiode efficiency	Moon et al. (2021)
Hardwired direct connection	Very high	The length of interconnecting wires	Not applicable	Infection, micromotion, neural apoptosis, patient death	Cable length, mobility	Sterilization, antimicrobial materials	Prasad et al. (2012), Potter et al. (2013), Willett et al. (2023)

### 3.1 Electromagnetic methods

#### 3.1.1 Inductive coupling

##### 3.1.1.1 Principles

Inductive coupling employs a dual-coil system: an external primary coil and an internal secondary coil implanted within the body. The primary coil generates a fluctuating magnetic field that induces voltage in the internal coil, enabling wireless power transmission to the implant (Sanghera, 2007). This technology is particularly well-suited for medical devices requiring high data rates and computational power, such as brain and spinal cord stimulators. For instance, a study by Lyu et al. developed a miniaturized stimulator measuring 5 mm × 7.5 mm in size, operating at a resonant frequency of 198 MHz and capable of functioning at a 14 cm distance from the external transmitter. This compact stimulator uses stored energy to deliver its output stimulus, eliminating the need for a separate stimulation control circuit block (Lyu et al., 2018).

##### 3.1.1.2 Near-field communication

Near-Field Communication (NFC) represents a specialized subgroup of inductive coupling, operating predominantly around 13.56 MHz and tailored for short-range interactions, typically up to 10 cm. While it is categorized under the broader spectrum of RF communication in literature, NFC’s operational characteristics align it with inductive coupling which involves the transfer of energy through electromagnetic fields between two closely spaced coils. NFC stands apart due to its specific design for short-range, high-frequency communication and power transfer. This specialized design results in NFC devices generating weaker electromagnetic fields compared to broader RF technologies, whose radiation levels vary based on power and application (Stoecklin et al., 2020; Lathiya and Wang, 2021; Van Mulders et al., 2022).

Moreover, NFC’s capability to support two-way data communication between an external coil or loop antenna and a second implanted coil, while penetrating the tissue barrier, further sets it apart. This dual functionality for both power transfer and data exchange at close proximity makes NFC a versatile choice for neural implant systems, serving specific purposes that broader inductive coupling technologies might not fulfill (Stoecklin et al., 2020; Lathiya and Wang, 2021; Van Mulders et al., 2022).

**Figure 2:**
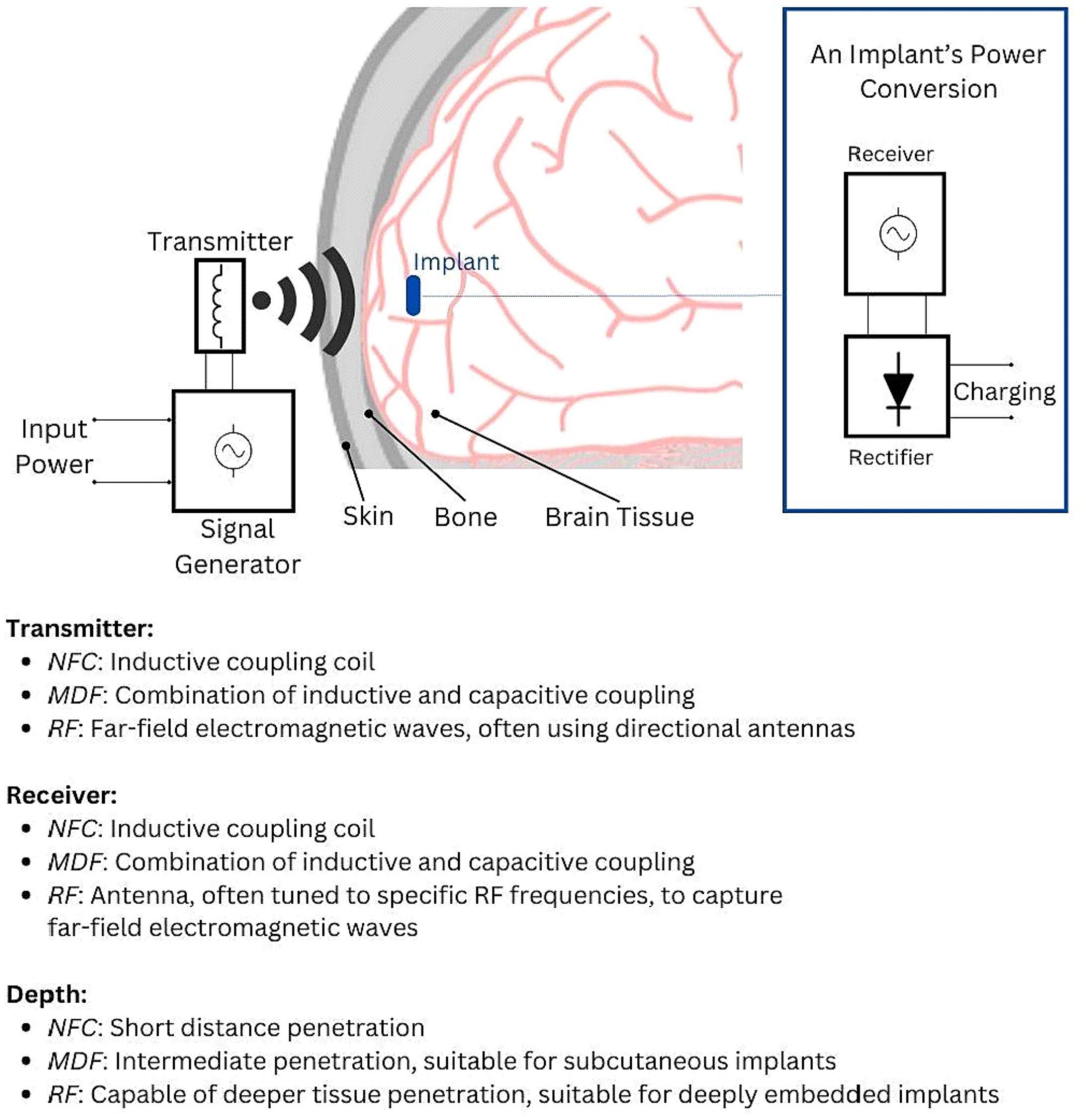
Illustration of the electromagnetic power transfer principle, showcasing how input power is converted into electromagnetic waves that traverse various media (air, skin, bone, and brain tissue). The implant’s power management circuitry receives this signal, converts it to an AC signal, and then rectifies it into DC voltage for charging the implant’s battery or capacitor. The key distinctions between NFC, MDF, and RF lie in their transmission and reception mechanisms. These differences influence wavelength, range, and depth of penetration, defining each technology’s unique operational characteristics.

Comparatively, Midfield Transfer (MDF) and Radio Frequency (RF) technologies, while sharing the basic principle of wireless energy transfer with inductive coupling, operate over different ranges and with varying efficiencies (Figure 2). MDF occupies an intermediate range, offering a compromise between the close proximity required by NFC and the longer reach of RF (Ho et al., 2014; Keerthi et al., 2018). RF, suited for longer-range power transfer, becomes less efficient over the short distances where NFC excels. NFC’s need for close proximity between the transmitter and implant, while potentially limiting in terms of positioning, is beneficial in minimizing electromagnetic exposure and ensuring targeted energy transfer (Freudenthal et al., 2007; Keerthi et al., 2018). A study showcased a Wireless Power Transfer (WPT) system, operating at 7.15 MHz, tailored for optogenetics. It featured a compact receiver with a micro-LED, essential for neuronal stimulation. Computational simulations yielded a return loss (S11) of −15.37 dB at the resonance frequency. The system could induce 500 mVpp to the receiver module from a separation of 5 mm, with an input power set at 0 dBm (Biswas et al., 2018).

##### 3.1.1.3 Challenges and optimization

Improper calibration can compromise the mutual inductance between the internal and external coils, reducing power transfer efficiency (Mahmood et al., 2022). Such inefficiency can result in the device overheating, posing risks of tissue damage (Yu et al., 2020). Misalignment between the coils can further disrupt the continuity of the RF signal, potentially leading to data loss and reduced operational capacity of the device (Liu et al., 2014). These issues are especially critical for deep-tissue implants, where calibration errors further limit the depth of energy transfer (Ho et al., 2014).

One avenue for optimization focuses on the coupling coefficient k, a measure of energy transfer efficiency ranging from 0 to 1. A value closer to 1 not only maximizes energy transfer but also minimizes potential thermal damage to neural tissues—a concern for BMIs requiring high computational power for real-time neural decoding (Haerinia and Shadid, 2020; Khan et al., 2020). Recent advances in circuitry have incorporated machine learning algorithms for predictive analytics on power needs optimizing efficiency (Huang et al., 2018).

###### 3.1.1.3.1 Eddy current losses and biological impact

The operating frequency of alternating magnetic fields is typically chosen between 100 and 1 MHz. This range minimizes eddy current losses, thereby reducing the risk of localized heating and its subsequent influence on neural stability (Maaß et al., 2017; Haerinia and Shadid, 2020; Mahmood et al., 2022). In addition to this, magnetic fields at these frequencies have the potential to disturb intracellular ionic concentrations, which can indirectly affect neural activity and cellular homeostasis (Kletetschka et al., 2021; Panagopoulos et al., 2021). Notably, a study by Zheng et al. (2017) demonstrated that even at a much lower frequency—specifically, a 15 Hz square wave magnetic field—there were alterations in the activation kinetics of sodium and potassium channels in cortical pyramidal neurons. Pall (2013) compared different electromagnetic field frequencies, noting their potential to activate voltage-gated calcium channels, leading to varied effects. Some frequencies may produce beneficial or adverse outcomes. This study emphasizes the importance of carefully evaluating the biological impacts of these frequencies, though not all ranges are directly related to the power transfer in brain implants.

###### 3.1.1.3.2 Distance between transmitter and receiver

In line with these challenges is the critical factor of the distance between the transmitter and receiver. This distance varies depending on several variables such as design specifications and operating frequencies. Inductive coupling requires relatively short distances between the transmitter and receiver for optimal power transfer (Singer and Robinson, 2021). For example a study on Printed Spiral Coils (PSCs) tailored for intracranial neuroprosthetics with PSCs optimized for a 10 mm face-to-face distance achieved varying efficiencies depending on the environment: 72.2% in air, 51.8% in saline, and 30.8% in muscle tissue. The study emphasized that the implant’s surrounding tissue significantly influences power transfer efficiency (Jow and Ghovanloo, 2009). Another study, using overlapping arrays of transmitter coils demonstrated 68% power efficiency at a 4 cm distance, highlighting the potential for maintaining consistent power in implants subject to motion (Pahlavan et al., 2022). Thus, meticulous calibration and design are imperative for optimizing both energy transfer and safety metrics, especially in the context of deep-tissue implants.

##### 3.1.1.4 Safety considerations

In the context of inductive coupling, particularly for brain implants, safety considerations are focused on the specific interaction of electromagnetic fields within the confines of the human body. Given that inductive coupling relies on closely spaced coils to transfer energy, managing the intensity and frequency of the electromagnetic fields is crucial to prevent any adverse thermal effects on surrounding brain tissues. As previously discussed in the first chapter, the Specific Absorption Rate (SAR) is a crucial safety metric, setting limits to mitigate the risk of thermal damage to neurons. Regulatory agencies like the FDA also mandate standards for electromagnetic disturbances, impacting device biocompatibility requirements (Adibzadeh et al., 2018; Review of published literature between 2008 and 2018 of relevance to radiofrequency radiation and cancer, 2020).

#### 3.1.2 Capacitive coupling

##### 3.1.2.1 Principles

Capacitive coupling utilizes electric fields to transfer energy, as opposed to inductive coupling, which relies on magnetic fields. The system comprises a transmitter and a receiver. The transmitter features filters to eliminate unwanted harmonics, a rectifier for AC/DC conversion, and an inverter that generates high-frequency AC power to excite the transmitter plate (Al-Saadi et al., 2018).

In active operation, opposite charges on adjacent plates of the transmitter create an alternating electric field, facilitating power transfer to the receiver. The receiver then converts the received AC power back to DC, making it suitable for biomedical implants (Al-Saadi et al., 2018). One study demonstrated power transfer capabilities through a 5 mm layer of biological tissue (beef) between two 3 cm square plates, employing Class E zero voltage switching to generate an alternating current at a 1 MHz frequency, offering solutions to several limitations inherent in the traditional bipolar CPT method (Al-Saadi et al., 2018).

Capacitive coupling has evolved to include wireless power transfer, initially finding applications in wideband data telemetry. Emerging research has explored its suitability for wireless powering of biomedical implants (Grob et al., 2016; Mustapa et al., 2018). Recent studies have examined its potential for wireless powering, where a pair of conductors is positioned on each side of the skin, separated by a distance D, and connected to an implant device with a load resistance RL. This system utilizes a closed current loop and relies on the displacement current, IDisp., between the conductor plates to establish wireless power transfer across tissue layers (Khan et al., 2020).

##### 3.1.2.2 Challenges and optimization

Despite its advantages, such as reduced eddy current loss and simplified system architecture, it requires a sufficiently large capacitor for energy storage and may risk electrical interference with other devices (Wang Z. A. et al., 2022). A recent study investigated link efficiencies and potential biohazards, revealing that capacitive coupling decays more slowly in power link efficiency as a function of plate distance compared to inductive coupling. Specifically, at a transmitted power of 1 W and a frequency of 5 MHz, capacitive coupling resulted in a 10-g averaged SAR (Specific Absorption Rate) value of 1.63 W/kg, lower than the 2.39 W/kg seen in inductive coupling of similar dimensions (Al-Kalbani et al., 2014).

###### 3.1.2.2.1 Parameter optimization

The efficiency and safety of capacitive coupling are influenced by key parameters like coupling capacitance Cm crucial for energy storage, and the coupling coefficient *k*, which quantifies the efficiency of the electric field between the transmitter and receiver. Optimization of these parameters not only enhances power transfer but can also reduce the SAR, thereby improving the system’s overall safety profile. Material selection and dimensional considerations, especially for coaxial cables, are also a critical factor for minimizing high-frequency eddy current losses (Al-Saadi et al., 2018).

###### 3.1.2.2.2 Frequency range

Beyond SAR, it is crucial to consider the operating frequency range, which for implant intra-body communication (IBC) is typically between 3 and 10 MHz. However, this range can vary depending on the specific application and circuit design, further emphasizing the need for optimal parameter selection (Zhang et al., 2014).

###### 3.1.2.2.3 Distance between transmitter and receiver

While capacitive coupling offers lower electromagnetic interference, it has limitations in power transfer efficiency (PTE) and is generally more suitable for short-range applications. However, advancements in the field have led to the development of Resonant Capacitive-Coupling (RCC) methods. In applications like intracranial pressure sensors, RCC methods have achieved PTEs of 34.14%, and even 42.21% when an additional intermediate plate is used (Narayanamoorthi, 2019). According to the same study capacitive plates used for power transfer can also facilitate data transmission, eliminating the need for a separate antenna. This is achieved using amplitude phase-shift keying (ASK) modulation techniques. Furthermore, the system can be integrated with Internet of Things (IoT) modules for remote health monitoring (Narayanamoorthi, 2019).

##### 3.1.2.3 Safety considerations

While SAR remains a crucial metric, the focus in capacitive coupling is also on the intensity of the electric field generated. It’s essential to ensure that the electric field strength remains within safe limits to prevent any adverse biological effects, such as neuronal irritation or damage (Laissaoui et al., 2017). Regulating SAR and electric field strength is key to minimizing the risk of thermal effects and ensuring that the energy transferred does not negatively impact the surrounding brain tissue (Griffiths et al., 1986).

#### 3.1.3 Far field antenna, radio frequency

##### 3.1.3.1 Principles

Radio Frequency (RF) technology employs far-field electromagnetic waves in the MHz and GHz range to wirelessly transmit energy and data over greater distances to brain implants differentiating it from the near-field magnetic fields utilized in inductive coupling and the intermediate-range operations of Midfield Transfer (MDF). Making it suitable for implants located deeper within the brain contrary to inductive coupling, more effective for superficial implants. An external transmitter generates these waves, captured by an antenna connected to an internal receiver in the implant, where it is converted to electrical power (Chow et al., 2013; Singer and Robinson, 2021). Incline inductive coupling, which predominantly uses magnetic fields, RF system uses magnetoelectric (ME) effects, enabling high power densities, tolerance to misalignment, and deep tissue penetration. These attributes make RF good for powering deep-located bioelectronic implants. Modulation techniques like amplitude (AM), frequency (FM), and phase (PM) are used for dual power and data transfer. An external “reader coil” generates the electromagnetic field, intercepted by a corresponding coil in the implant. The received signal is demodulated to separate power and data, effectively fueling and interfacing with the neural implant (Ferguson and Redish, 2011; Pavone et al., 2012).

##### 3.1.3.2 Challenges and innovations

###### 3.1.3.2.1 Distance between transmitter and receiver

One of the primary constraints in distance between the transmitter and receiver in Radio Frequency (RF) charging systems is the inherent limitation on the size of the receiver’s antenna and wavelength of the electromagnetic waves used which influences the effectiveness of energy transfer (Ferguson and Redish, 2011).

For optimal energy transfer, the dimensions of the antenna should ideally be commensurate with the wavelength of the RF waves. This requirement becomes challenging while using higher frequencies, where the wavelengths are shorter which ideally require smaller antennas. However brain implants require minimization of the system to reduce the invasiveness of the procedure which might conflict with the ideal antenna size needed for efficient RF energy transfer, leading to a trade-off between the physical size of the implant and the efficiency of power reception (Ferguson and Redish, 2011; Di Carlo et al., 2018; Fan et al., 2022).

Moreover, the distance factor in RF charging systems also influences the power efficiency. As the distance between the transmitter and receiver increases, the power transfer efficiency typically decreases due to the spreading of electromagnetic waves. This dispersion of energy means that only a fraction of the transmitted power is captured by the receiver, necessitating higher power outputs from the transmitter to ensure adequate energy reaches the implant. This increase in power output can, in turn, lead to heightened concerns about tissue heating (Di Carlo et al., 2018; Fan et al., 2022; Guo et al., 2022).

Addressing the distance challenge, initially targeted miniature, digestible sensors. However, these advancements have broader implications, including applicability to brain implants. The significant loss of radio wave power as they traverse biological tissues has been a major hurdle (Ma et al., 2018; Wireless system can power devices inside the body, 2018). To tackle this, researchers introduced *In-Vivo* Networking (IVN), a novel system based on beamforming algorithms. The IVN approach uses an antenna array to emit radio waves at varying frequencies. These frequencies overlap at specific points, intensifying the energy sufficiently to power deeply implanted devices (Ma et al., 2018; Wireless system can power devices inside the body, 2018). This method also eliminates the need for precise implant positioning, enabling the simultaneous powering of multiple devices. *In vivo* tests using pigs as a model have demonstrated the system’s robustness. Sensors located as deep as 10 cm within tissue could be powered from an external distance of up to 1 m. When the sensors were situated closer to the skin surface, this operational range extended to 38 m, shedding light on the trade-off between implant depth and external transmission range (Ma et al., 2018; Wireless system can power devices inside the body, 2018).

###### 3.1.3.2.2 Energy transfer efficiency

The efficiency of RF-based energy transfer is contingent on various parameters, including but not limited to, input power levels and device design. For instance, one study reported a maximum RF-to-DC conversion efficiency of 82% at an input power level of 2 dBm. However, the power transmission efficiency (PTE) was relatively low at 0.007%. Another reported an RF-to-DC efficiency of 42% at −10 dBm for skin-implanted devices, with PTE enhanced by the use of a high-permittivity dielectric layer on the body (Iqbal et al., 2022).

###### 3.1.3.2.3 Biomedical advances

Advancements in the biomedical field have also shown promise. One research initiative presented a bimodal implantable rectenna that operates at frequencies of 0.915 and 2.45 GHz, achieving radio frequency to direct current (RF-to-DC) conversion efficiencies of 79.9 and 72.8%, correspondingly. The dual-frequency antenna employed a meandered resonator design, coupled with an optimized rectification circuit, to minimize dimensions without compromising functionality. This rectenna’s antenna and rectifying elements were encapsulated within a capsule-sized device, measuring a mere 5 × 5.25 × 0.25 mm, highlighting its potential applicability in neural implant technologies (Iqbal et al., 2022).

#### 3.1.4 Mid field inductive coupling

##### 3.1.4.1 Principles

Mid Field Inductive Coupling (MDF) is a method of energy transfer that operates in the midfield range, between near-field (such as inductive coupling) and far-field (like RF energy transfer) techniques. The operating frequency for MDF is typically in the range of tens to hundreds of megahertz (Keerthi et al., 2018). This range is chosen to balance tissue penetration depth with safety and efficiency. The specific frequency chosen depends on the application, implant size, and the required power transfer distance. It employs electromagnetic waves that are larger than the device but smaller than the distance over which power is transferred. The basic components of an MDF system include a transmitter that generates electromagnetic waves, and a receiver, typically integrated within the implant, that captures these waves to extract energy (Ho et al., 2014; Keerthi et al., 2018).

In MDF, the transmitter generates electromagnetic fields at a frequency that allows the waves to effectively penetrate biological tissues without significant attenuation. Unlike inductive coupling, which is limited by the mutual inductance between closely spaced coils, MDF can transfer energy over larger distances relative to the size of the transmitter and receiver. This makes it particularly suitable for implants located deeper in the brain (Ho et al., 2014; Dinis et al., 2017; Keerthi et al., 2018).

##### 3.1.4.2 Challenges and innovation

###### 3.1.4.2.1 Miniaturization of receiver

The miniaturization of the MDF receiver is not just a matter of reducing size, but also of maintaining or enhancing performance in a smaller implant space. Research in this area is exploring ultra-compact antenna designs and the use of materials like graphene and metamaterials, which offer high conductivity and electromagnetic properties in extremely thin and flexible forms (Riaz et al., 2023; Saidi et al., 2023). The challenge also extends to integrating these miniaturized components into the implant without affecting its biocompatibility or durability (Dinis et al., 2017; Keerthi et al., 2018; Singer and Robinson, 2021).

###### 3.1.4.2.2 Alignment sensitivity

To tackle the issue of alignment sensitivity, there is a push toward the development of ‘smart’ MDF systems equipped with real-time alignment correction capabilities (Singer and Robinson, 2021). To enhance misalignment new approaches can employ multiple coil structures. The systems, such as the hybrid array resonator structure described in recent research, utilize arrays of coils to create a more uniform magnetic field, significantly improving efficiency despite misalignment (Wang et al., 2020).

##### 3.1.4.3 Safety considerations

In MDF systems, precise control of the SAR is crucial for safety, particularly given the brain’s sensitivity to temperature changes. MDF’s operation in an intermediate range poses challenges in ensuring uniform energy absorption due to the varied electrical properties of biological tissues. Effective SAR regulation is essential to prevent potential thermal effects in these systems (Truong et al., 2020).

Another aspect of safety is the long-term effects of exposure to electromagnetic fields generated by MDF. While the frequencies used in MDF are generally considered safe, the long-term impact of continuous exposure on biological tissues is still an area of active research as mentioned in the biocompatibility section (RăcuciU et al., 2015).

#### 3.1.5 Magnetoelectric

##### 3.1.5.1 Principles

The principles of magnetoelectric (ME) energy transfer in neural implants revolve around the interaction between magnetic and electric fields, often mediated through ultrasonic waves, to enable efficient and targeted energy delivery. This approach uses the magnetoelectric effect where magnetic fields can induce electric fields in certain materials, and vice versa (Kopyl et al., 2021).

The core of the ME system is the magnetoelectric transducer, which typically consists of a composite material that combines piezoelectric and magnetostrictive layers. The piezoelectric component generates an electric field in response to mechanical stress, while the magnetostrictive material responds to magnetic fields by changing shape or dimensions (Yu et al., 2020; Kopyl et al., 2021).

In practice, an external transmitter generates a magnetic field, which is often modulated by ultrasound waves. These ultrasound waves, with their ability to penetrate biological tissues efficiently, carry the magnetic field deep into the body where the implant is located. Upon reaching the transducer, the magnetic field causes the magnetostrictive material to undergo a deformation. This mechanical deformation is then converted into an electrical voltage by the piezoelectric component, effectively transferring energy from the external source to the implant (Dong et al., 2003; Zhai et al., 2006; Singer et al., 2020).

A practical example of ME energy transfer can be found in MagNI, a magnetoelectric neural implant designed for deep tissue stimulation (Yu et al., 2020). This proof-of-concept device wirelessly receives over 1 mW power through an ME power link, capable of reaching mm-sized implants implanted up to 30 mm dee MagNI features a low-frequency magnetic field (250 kHz) to minimize tissue absorption and reflection, enhancing power delivery efficiency. Despite its miniature size (8.2 mm^3^, 28 mg), it offers adaptive operation, 1-V source variation tolerance, and programmable bi-phasic current stimulation, demonstrating significant advancements in bioelectronic medicine.

##### 3.1.5.2 Challenges and innovations

###### 3.1.5.2.1 Optimizing magnetoelectric materials

One of the challenges is finding and optimizing materials that exhibit a strong magnetoelectric effect. This involves researching and developing composite materials that can efficiently convert magnetic energy into electrical energy at lower intensities. Innovations in materials science are key here, as the effectiveness of ME technology heavily relies on the properties of these materials. Researchers are experimenting with various combinations of piezoelectric and magnetostrictive materials to enhance the conversion efficiency while ensuring biocompatibility (Dong et al., 2003; Zhai et al., 2006; Singer et al., 2020).

###### 3.1.5.2.2 Energy conversion efficiency

Maximizing the energy conversion efficiency of ME systems is another significant challenge. This involves not only optimizing the materials but also the overall design of the transducer and the tuning of the magnetic field generator and ultrasonic modulation. Engineers and scientists are exploring various transducer geometries and configurations to enhance their energy conversion capabilities, ensuring that the implants receive sufficient power to function effectively (Iqbal et al., 2022; Marrella et al., 2023; Sasmal and Arockiarajan, 2023).

##### 3.1.5.3 Safety considerations

ME energy transfer technology, specific to neural implants, necessitates a focused approach to safety, primarily due to its utilization of the magnetoelectric effect. This effect involves converting magnetic energy into electrical energy within the implant, often mediated through ultrasonic modulation.

Specific Absorption Rate (SAR) is a key metric in assessing the safety of ME systems. This is because ultrasound, at high intensities, can pose risks such as tissue heating, necessitating careful calibration of the system (Bocan et al., 2017).

### 3.2 Acoustic methods

#### 3.2.1 Ultrasound

##### 3.2.1.1 Principles

**Figure 3:**
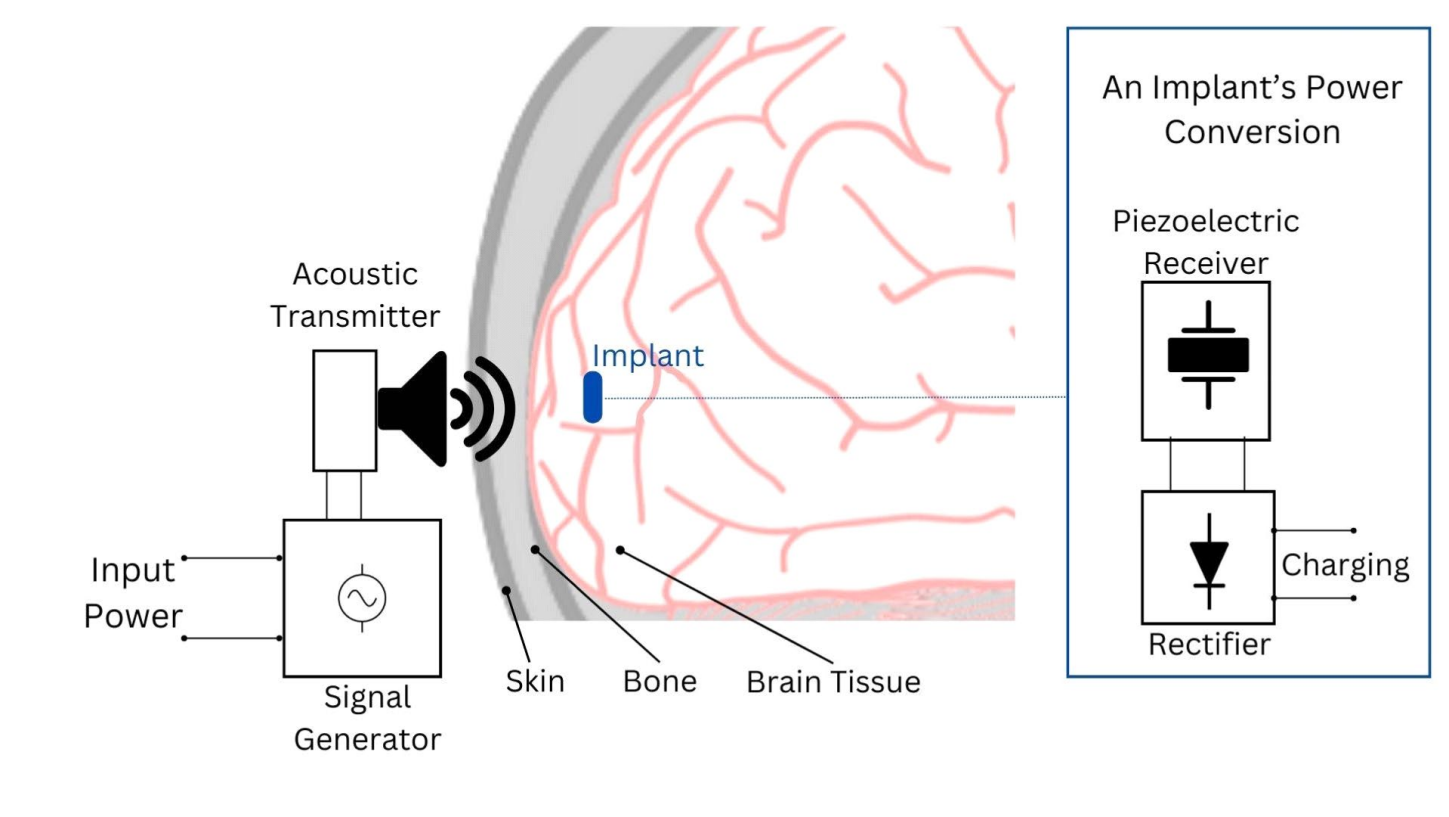
Depiction of the acoustic power transfer principle for brain implants. In this system, an acoustic transmitter, typically an ultrasound transducer, generates waves that penetrate to the implant’s location. The implant’s acoustic receiver, often a piezoelectric device, captures these waves and converts them into electrical energy. This energy is then rectified into DC voltage to power the implant’s circuitry or charge its battery/capacitor.

Ultrasound employs high-frequency acoustic waves to facilitate wireless power transfer between an external and an internal transducer. The external transducer emits focused ultrasonic waves targeting a piezoelectric material connected to the internal transducer inside the skull (Figure 3). This material converts the mechanical energy from the ultrasound waves into electrical energy, which powers the brain implant (Jiang et al., 2020; Athanassiadis et al., 2021; Birjis et al., 2022).

The frequency range for ultrasonic powering is application-dependent and must be carefully selected to optimize factors such as tissue attenuation, Rayleigh distance, and the dimensions of both the receiver and transmitter and their distance. Operating the transducer close to its resonance frequency is crucial for maximizing power transfer, as the resonance frequency depends on the geometry and material of the transducer. Although ultrasonic power transfer has lower tissue absorption compared to other techniques like RF approaches, it is crucial to consider the absorption of ultrasonic energy in tissue to avoid exceeding the specific absorption rate (SAR) as an increase in frequency can lead to increased tissue absorption losses due to an extended Rayleigh distance (Basaeri et al., 2016). For example Ultrasound operates at dramatically lower frequencies in tissue, which results in reduced wavelength compared to EM.

##### 3.2.1.2 Challenges and innovations

###### 3.2.1.2.1 Distance and efficiency

Rayleigh distance is a critical factor in the efficiency of ultrasound power transfer, marking the point where the ultrasound beam is naturally focused and power density is optimal. Beyond this distance, efficiency decreases as the beam diverges, though the system can still function. For example, a study at UC Berkeley highlighted that for 10 MHz ultrasound in brain tissue, the wavelength λ = 150 μm, while for 10 GHz EM, λ = 5 mm, suggesting the receiver should ideally be at one Rayleigh distance from the transmitter for maximum efficiency, especially in miniaturized applications (Seo, 2013).

Comparatively, ultrasonic links have shown superior performance over inductive links in powering small, deeply implanted receivers. A study revealed that for receivers of 1.1 mm^3^, the ultrasonic link achieved a Power Transmission Efficiency (PTE) of 0.65% at an optimal frequency of 1.1 MHz, significantly higher than the inductive link’s PTE of 0.05% at 30 MHz. However, ultrasonic links are vulnerable to receiver (Rx) misalignments and orientations, posing design constraints. For larger receivers of 20 mm^3^ at depths less than 30 mm, inductive links surpass ultrasound in PTE and PDL (Ahmed et al., 2018).

Recent innovations aim to simplify beamforming in ultrasonic systems, traditionally reliant on complex electrically controlled phased arrays. A study introduced the Sectored-Multiring Ultrasonic Transducer (S-MRUT), built on a single piezoelectric sheet, capable of focusing ultrasonic waves on implants at various depths without the complexity of phased arrays (Hosseini et al., 2021). This advancement presents a promising solution for selectively powering brain implants and effectively penetrating tissues (Ultrasonically powered implantable devices, n.d.).

###### 3.2.1.2.2 Depth of operation and miniaturization

Further, buttressing ultrasound’s potential are advances in miniaturization and operational depth. For example researchers at Stanford University featured an implant with dimensions of 4 mm × 7.8 mm that operated at an incident acoustic intensity within 5% of the FDA diagnostic limit. This implant supported a DC load up to 100 μW, suggesting the feasibility for higher available DC power levels between 100 μW to a few mWs (Charthad et al., 2015). In a subsequent study by the same group, an implant of 2 mm × 3 mm × 6.5 mm dimensions achieved a remarkable depth of operation—10.5 cm in a tissue phantom. This depth is significantly greater than what current technologies offer, and it maintains a wide range of stimulation parameters, from 22 to 5,000 μA in amplitude and 14 to 470 μs in pulse-width (Charthad et al., 2018).

##### 3.2.1.3 Safety considerations

Ultrasound, widely utilized in various medical applications, is generally considered safe due to its non-ionizing nature (Seo et al., 2015; Rosa and Yang, 2020). The application of ultrasound in powering brain implants introduces safety considerations concerning the intensity of the acoustic waves. Unlike its use in imaging, wireless power transfer often requires higher intensities to transmit energy effectively through biological tissues. At these elevated intensities, ultrasound can potentially cause localized heating depending on the tissue structure. This risk arises from the interaction of ultrasound waves with tissues, where the vibration of molecules, especially in denser tissues, can lead to an increase in temperature (Tel Haar, 2011; Proto et al., 2022).

### 3.3 Optical methods

#### 3.3.1 Optical-charging

##### 3.3.1.1 Principles

Optical charging is an emerging yet under-researched method for powering brain implants. This technology capitalizes on the versatility of photons, which can travel freely through the air. An external laser directs focused light beams toward an integrated photovoltaic cell, which consists of a p-n junction made from a large-band-gap semiconductor. This setup efficiently converts incoming light into electrical power for the implant (Park et al., 2015, 2021). The wavelength of light is usually selected to be in the near-infrared (NIR) range, owing to its deeper penetration into biological tissues (Hososhima et al., 2015). For instance, research at Osaka University has shown that 810-nm wavelength NIR light could effectively charge a lithium secondary battery embedded in the implant, providing a direct power supply (Goto et al., 2001). Unlike RF beams, laser beams in optical charging systems can sustain their size and power across longer distances. However not yet used for powering implanted implants this attribute suggests that the transmission efficiency of a mono-wavelength laser remains relatively stable regardless of the increase in distance (Ding et al., 2020). In contrast, Radio Frequency (RF)-based charging exhibits suboptimal efficiency at lower frequencies. Elevating the frequency, however, precipitates concerns such as tissue absorption, establishing a trade-off: lower frequencies engender decreased efficiency, while higher frequencies foster adverse tissue absorption. In a study incorporating a self-powered mote with NIR wireless power and data transfer, an implant showing promising results in a simulated brain environment is designed (Moon et al., 2021). A fiber-optic Y-probe has been used in experimental setups to simultaneously illuminate a PV cell at 850 nm for power delivery and a 1,000 nm LED for near-infrared data exchange. This effectively shows that power and data may be wirelessly delivered while simulating the link conditions between the mote and receiver. The projected −41.3 dB total losses of such an experimental optical link point to the necessity for additional improvement. The implementation of neural motes and the repeater unit system still requires further work, even if this work illustrates the basic functioning principles. Additionally, the use of such lasers does not induce electromagnetic interference (EMI) in existing radio communications, providing another advantage over RF-based systems (Ding et al., 2020).

##### 3.3.1.2 Challenges and optimization

###### 3.3.1.2.1 Distance

Optical charging faces challenges related to the effective transmission distance, which is influenced by various factors such as tissue opacity, scattering, and absorption. However, NIR light appears to mitigate some of these issues (Hososhima et al., 2015). For example, a comparative study suggests that an energy transfer efficiency above 0.7 can be achieved within a distance of 650 cm when a reflector is used, significantly extending the effective distance of this technology (Ding et al., 2020).

###### 3.3.1.2.2 Energy transfer efficiency

Efficiency in optical charging is determined by various factors including the photodetector’s efficiency, light scattering, absorption by tissues, and alignment between the external and internal units. Optical methods present a compelling alternative, particularly when the implant is situated deep within tissues where traditional electromagnetic interference is a concern (Ding et al., 2020). For instance, using a photodiode area of 2.1 roughly 17 min of NIR light exposure at a power density of 22 mW/cm was sufficient to power a commercial cardiac pacemaker for 24 h (Goto et al., 2001). Another study focused on cardiac pacemakers utilized a 5 mW laser emitting at a 750 nm wavelength to charge a 150 mAh LiPo battery embedded under the skin. The approach regulated the implant’s power to below 10 mW, and a 60-min charge could sustain the battery for 85 h (Iqbal et al., 2019). However, misalignment of the receiver photodiodes with the transmitter can significantly reduce Power Transfer Efficiency (PTE), emphasizing the need for precise alignment (Ding et al., 2020). A study indicates that 670 nm NIR light penetrates 2.5 mm into live tissues and has high transmittance (60–70%), boosting energy transfer efficiency. Conversely, 450–600 nm wavelengths have lower transmittance (30–50%), limiting their efficacy (Kim et al., 2020).

###### 3.3.1.2.3 Data transfer

Optical charging methods can also facilitate bidirectional data transfer. Here, the intensity, frequency, or phase of the light beam can be modulated to encode data. Just as in power transfer, limitations regarding distance and tissue absorption also apply to data transfer.

##### 3.3.1.3 Safety considerations

The foremost safety concerns in optical charging pertain to the thermal effects that result from tissue absorption of light. While some studies have recorded a modest temperature rise of 1.4°C in the skin during light irradiation (Goto et al., 2001) others found no noticeable side effects or temperature changes during a 60-min charging period for cardiac pacemakers. These findings hint at the method’s potential for safe use in controlled biomedical environments (Iqbal et al., 2019).

#### 3.3.2 Direct connection to the outside body

##### 3.3.2.1 Principles

Direct hardwired connection in brain implant technology primarily employs a specialized cable linking the implant to an external power source. This solution usually involves Intracortical microelectrodes. This method is particularly beneficial for high-power applications and in pre-clinical trials. In *in-vivo* electrophysiology studies, similar direct connections are used, where electrode arrays are implanted in rodents and primates. Conventionally, hardwired implants were presented as battery-less electrodes powered from outside, which was limiting patients’ mobility (Bechtereva et al., 1975). However, with the evolution of the technology a hardwired approach is needed to recharge the embedded battery of an implant. Rechargeable hardwired brain implants, connected to an external power source via a specialized cable, offer the to be recharged without surgical intervention (Ewing et al., 2013; Waln and Jimenez-Shahed, 2014).

A wired connection typically demonstrates higher efficiency than a wireless connection using a transmitter and receiver. In wireless systems, power loss often occurs due to the physical layout of the system and impedance mismatches at various junctions, including the air and tissue interface. To mitigate these losses, wireless systems might need increased power output, but this is constrained by safety regulations to avoid excessive heat or electromagnetic field exposure, ultimately affecting the maximum usable power for the implant (Singer and Robinson, 2021).

##### 3.3.2.2 Energy transfer efficiency

Direct connection methods are widely used in various neural implant systems. For example, the BrainGate system transitioned to a wireless connection and a small transmitter; it utilized a Utah Array implanted in the brain, featuring 100 micro-electrodes capable of interacting with up to four neurons. This array was tethered via a cable to an external computer for signal processing and power, ensuring a stable and continuous energy supply to the implant. While BrainGate initially required a more cumbersome cabling system, both companies have since transitioned toward wireless technologies, with BrainGate even demonstrating the first human use of a high-bandwidth wireless system (Bullard, 2019; Juskalian, 2020; Woeppel et al., 2021; BrainGate, 2022). Similarly, Neuralink’s approach to system’s testing in development stages before implementing wireless solutions. Neuralink relied on an implant with up to 3,072 channels across 96 threads, powered through a USB-C cable that also allowed for full-bandwidth data streaming. Neuralink designed its system to be both scalable and low-power, incorporating a specialized charger with an aluminum battery base and a detachable remote coil for added charging flexibility (Musk and Neuralink, 2019).

##### 3.3.2.3 Challenges and optimization

###### 3.3.2.3.1 Cable distance and mobility restrictions

Unlike wireless methods that may have distance limitations based on factors like the Rayleigh distance, direct connections do not inherently have such restrictions. However, the length of the cable can be a limiting factor in terms of mobility and comfort both for human subjects, rodents or primates affecting natural behavior of animals in experiments thus potentially affecting the results (McGlynn et al., 2021).

##### 3.3.2.4 Safety concerns

While effective, the direct connection poses heightened risks, particularly concerning infection due to the breach of the body’s natural barriers requiring rigorous sterilization protocols and may use antimicrobial materials to mitigate risks. Moreover, there are concerns that wired connections can affect micromotion inside the brain (McGlynn et al., 2021) This not only compromises the quality and consistency of the data collected but also poses a risk of physical damage to the brain tissue. Intracortical microelectrodes, used in applications ranging from recent advances in speech-to-text with ALS patients (Willett et al., 2023), allowing primates to play pong (Play Studio, n.d.) to clinical trials for motor deficit treatments (Gilja et al., 2015) face a decline in recording quality over time (Chestek et al., 2011; Jorfi et al., 2014; Kozai et al., 2015). Other concerns among others relate to oxidative stress as a response to microelectrode (Nguyen et al., 2016) presence of proinflammatory cells such as activated microglia, macrophages, and astrocytes (McConnell et al., 2009; Ravikumar et al., 2014) which could lead to neural apoptosis, further infection or patient death as outcome of further complications (Prasad et al., 2012; Potter et al., 2013).

## 4 Conclusion

The development of biocompatible neural interfaces is a complex and interdisciplinary field that requires expertise in neurobiology, materials science, and engineering. This paper discussed one of the critical components standing in the way of better implants: finding a way to power the implant while considering material durability, metabolic energy requisites, spatial limitations, and risk mitigation strategies.

Several methods for delivering power to neural implants were discussed, each with its unique set of challenges and states of research maturity. Electromagnetic methods like inductive and capacitive coupling, the most researched ones, offer the benefit of wireless energy transfer but come with their own limitations related to efficiency and tissue heating. Ultrasound techniques were also considered, showing promise in power transmission efficiency and multi-node interrogation capabilities with multiple research already incorporating this solution. Optical methods appear promising but still need to be well-studied to ensure patient safety, especially regarding potential tissue absorption. Direct connections to the outside body, while efficient, pose substantial risks such as infection and micromotion disturbances within neural tissue.

Careful consideration of an interdisciplinary approach is essential to foster innovative solutions to end-to-end problems without creating new ones while implementing brain implants. Moving forward, technological advancements must focus on mitigating existing limitations in the realms of biodegradability, biocompatibility, and minimized surgical interventions. Future research should delve into alternative mechanisms for energy transfer and explore materials that can be biologically integrated with the neural architecture or even bioengineer alternative solutions to address neurological conditions while considering discussed safety measures. Moreover, statistical models and computational simulations could serve as invaluable tools in predicting means of power, its performance, biocompatibility metrics, and risk factors such as heat dissipation or potential interference of frequencies both on the microbiological side and engineering side, thereby aiding in the pre-clinical phases of development. Furthermore, intelligently integrating with the neural system, adapting to the dynamic neural environment and responding in real-time to changes in neurological conditions to increase the energy efficiency, optimizing implants’ functionality with minimal risk.

## Author contributions

SM: Writing – original draft, Writing – review & editing. WP: Writing – original draft, Writing – review & editing. NH: Writing – original draft, Writing – review & editing.
